# Tissue non-specific alkaline phosphatase production by human dental pulp stromal cells is enhanced by high density cell culture

**DOI:** 10.1007/s00441-014-2106-3

**Published:** 2015-02-01

**Authors:** Matthew J. Tomlinson, Caitriona Dennis, Xuebin B. Yang, Jennifer Kirkham

**Affiliations:** 1 Department of Oral Biology, School of Dentistry, University of Leeds, Leeds, LS9 7TF UK; 2 NIHR Leeds Musculoskeletal Biomedical Research Unit, Chapel Allerton Hospital, Leeds, LS7 4SA UK

**Keywords:** Tissue non-specific alkaline phosphatase, Dental pulp, DPSC, Cell density, Flow cytometry

## Abstract

The cell surface hydrolase tissue non-specific alkaline phosphatase (TNAP) (also known as MSCA-1) is used to identify a sub-population of bone marrow stromal cells (BMSCs) with high mineralising potential and is found on subsets of cells within the dental pulp. We aim to determine whether TNAP is co-expressed by human dental pulp stromal cells (hDPSCs) alongside a range of BMSC markers, whether this is an active form of the enzyme and the effects of culture duration and cell density on its expression. Cells from primary dental pulp and culture expanded hDPSCs expressed TNAP. Subsequent analyses revealed persistent TNAP expression and co-expression with BMSC markers such as CD73 and CD90. Flow cytometry and biochemical assays showed that increased culture durations and cell densities enhanced TNAP expression by hDPSCs. Arresting the hDPSC cell cycle also increased TNAP expression. These data confirm that TNAP is co-expressed by hDPSCs together with other BMSC markers and show that cell density affects TNAP expression levels. We conclude that TNAP is a potentially useful marker for hDPSC selection especially for uses in mineralised tissue regenerative therapies.

## Introduction

The alkaline phosphatase (ALP) hydrolases are a group of three isoenzymes responsible for the dephosphorylation of a variety of molecules associated with a range of biological processes (Moss 1982). Of the three ALP isoforms in humans, two—intestinal and placental—are tissue specific. The third, tissue non-specific ALP (TNAP), is found in many tissues including bone, liver and kidney (Mckenna et al. 1979). ALPs are cell surface glycoproteins but can also be found in serum as a result of cell death and as such they are useful diagnostic biomarkers for a range of diseases including metastatic cancers and Paget’s disease of bone (Brown et al. 2005; Cathala and Brunel 1975; Jaffe and Bodansky 1943; Price et al. 1980). ALP is also used in in vitro assays as a marker of differentiation, especially in osteogenic cell cultures (Matsui et al. 1992; Sudo et al. 1983). Recent reports using human bone marrow stromal cells (BMSCs) have described the use of TNAP as a selective marker for the isolation of BMSCs from a mixed population (Battula et al. 2009). These authors described TNAP as “mesenchymal stem cell antigen-1 (MSCA-1)”; however, further studies confirmed that TNAP and MSCA-1 are the same antigen (Sobiesiak et al. 2010). We will therefore refer to TNAP throughout this paper.

In dental tissues, TNAP is expressed by ameloblasts, odontoblasts and stratum intermedium cells of the dental pulp (Hoshi et al. 1997; Hotton et al. 1999; Lezot et al. 2006). This expression is likely to be related to the role of these cells in enamel and dentine biomineralisation, where availability of phosphate ions is essential for hydroxyapatite nucleation and secondary crystal growth. However, what is not known is the extent to which human dental pulp stromal cells (hDPSCs) express TNAP. hDPSCs, first described in 2000 by Gronthos et al. are resident in low numbers in the dental pulp but are capable of mass expansion upon induction in in vitro culture (Gronthos et al. 2000). hDPSCs are of the mesenchymal lineage, capable of plastic adherence, colony formation and in vitro differentiation toward the dentinogenic, chondrogenic and myogenic lineages, as well as expressing the cell surface markers CD73, CD90 and CD105 (Dominici et al. 2006; Gronthos et al. 2002; Zhang et al. 2006). Previously, expression of ALP by cultured hDPSCs has been determined biochemically and used as a marker for the cells’ ability to produce mineralised matrix in vitro (Gronthos et al. 2000; Luisi et al. 2007; Wei et al. 2007).

Following the discovery of hDPSCs and confirmation of their potential to form a mineralised matrix, they have been seen as a potential source of cells for dental tissue engineering as well as an alternative cell source for use in reparative orthopaedic procedures (D’aquino et al. 2008; Gronthos et al. 2002; Krebsbach and Robey 2002). This is due to their relative ease of access for banking (e.g., from the deciduous dentition) as well as their mineralisation potential in vitro (El-Gendy et al. 2013; Gronthos et al. 2000; Karaöz et al. 2010; Pivoriūnas et al. 2009). However, low tissue volume and subsequent low cell numbers limit hDPSC translational utility, as ex vivo expansion is required in order to achieve clinically significant cell numbers for regenerative therapies (Bianco and Robey 2001; Guillot et al. 2007). Cell expansion can lead to potential problems, such as telomere shortening, changes to gene expression profiles and a reduction in mineralisation potential, which is a cause for concern for re-implantation strategies (Mokry et al. 2010; Zhang et al. 2008). A further potential problem with in vitro expansion of cells for transplantation is that of possible cell heterogeneity. One way to partially overcome this is to use cell separation technologies to isolate populations of cells that are likely to be clinically useful. For example, for dental and orthopaedic repair, it would be potentially beneficial to isolate cells that are predisposed to differentiate toward a mineralising phenotype. However, for this to be achieved, candidate cell surface markers must first be identified, as currently markers that can be used to predict the mineralisation potential of hDPSCs are scarce. TNAP is one such candidate marker.

The aim of this work is to increase our understanding of TNAP expression by hDPSCs. Our hypothesis was that, as TNAP is expressed by BMSCs, then as phenotypically similar cells, hDPSCs should also express this marker. hDPSC expression of TNAP should therefore map to the stromal cell compartment and be maintained throughout cell culture. In this report, we describe the expression of TNAP by cultured hDPSCs, co-expression of TNAP with dental stromal cell markers and modulation of expression by cell density and proliferation potential.

## Methods

### Cell extraction and isolation

hDPSCs were isolated from extracted impacted third molars obtained with patient consent through the Leeds Dental Institute Research Tissue Bank (07/H1306/93+5). Donors were both male (5) and female (7) and 18–38 years of age (*n* = 12). Tooth surfaces were cleaned with 70 % (v/v) ethanol-soaked tissue paper and any attached soft tissue was removed with a sterile scalpel blade. Teeth were fractured to allow exposure of the dental pulp, which was gently removed from the tooth fragments with sterile forceps. Extracted pulps were mechanically disrupted with a scalpel blade and digested in a solution of 3 mg/mL collagenase type I (Sigma-Aldrich, Poole, UK) and 4 mg/mL dispase (Roche, Mannheim, Germany) for 30 min at 37 °C with gentle agitation on a MACSmix tube rotator (Miltenyi Biotec, Bergisch Gladbach, Germany). The resulting suspension was passed through a 70-μm filter (BD Biosciences, San Jose, CA, USA) and centrifuged at 200*g* for 5 min before resuspension of the cell pellet in alpha modification of Eagle’s medium (α-MEM) (Life Technologies, Paisley, UK) supplemented with 15 % FCS (Biosera, Ringmer, UK), 2 mM L-glutamine (Sigma-Aldrich) and 100 units/mL penicillin/100 μg/mL streptomycin (Sigma-Aldrich). Resuspended cells were incubated in T25 flasks (Corning, Amsterdam, Netherlands) at 37 °C in 5 % CO_2_ in air at a ratio of 1 digested pulp per flask for 10–14 days or taken directly for flow cytometry. Human gingival fibroblasts (hGFs) were isolated from gingival tissue attached to the same third molar teeth used for pulp isolations. The tissue was removed from the tooth with forceps and subsequently mechanically disrupted with a scalpel blade before tissue fragments were plated into T75 flasks and cultured in α-MEM containing 10 % FCS, 2 mM L-glutamine and 100 units/mL penicillin/100 μg/mL streptomycin at 37 °C in 5 % CO_2_ in air for 10–14 days to allow for hGFs to adhere and proliferate.

### Cell culture

Digested pulps were cultured for 10–14 days before assessment of colony formation. Subconfluent flasks were passaged by digestion with 0.25 % trypsin/0.02 % EDTA (Sigma-Aldrich) and the resulting suspension was transferred to a sterile T175 flask at a density of 5 × 10^3^ cells/cm^2^; this flask was designated as p1. Passaged cells were subsequently cultured in basal medium of α-MEM containing 10 % FCS, 2 mM L-glutamine and 100 units/mL penicillin/100 μg/mL streptomycin at 37 °C in 5 % CO_2_ in air until 80 % confluency. Subsequent passages were performed as previously described. The same regimen was utilised for hGFs and BMSCs (Lonza, Slough, UK).

### Time course and density cultures

hDPSCs of p2–p4 from 5 donors were seeded to 6-well plates and cultured in basal medium at 37 °C in 5 % CO_2_ in air for varying times and at varying densities. To investigate the effect of time on TNAP expression by hDPSCs, cells were cultured for 14 days at an initial seeding density of 5 × 10^3^ cells/cm^2^. BMSCS were similarly cultured and analysed using the same methods. To determine the effect of cell density on TNAP expression, hDPSCs were cultured for 1 week with initial seeding densities ranging from 5 × 10^3^–1 × 10^5^ cells/cm^2^ with an initial change of medium performed 24 h after seeding to remove unattached cells. Upon termination of the culture periods, the cells were characterised by flow cytometry and specific staining.

### Mitomycin C culture

Subconfluent p2–p4 hDPSCs from 5 donors were passaged using 0.25 % trypsin/0.02 % EDTA, plated to T75 flasks at a density of 5 × 10^3^ cells/cm^2^ and cultured for 24 h to allow cellular adhesion. After 24 h, the basal medium was supplemented with 20 μg/mL mitomycin C (Sigma-Aldrich) to inhibit cell proliferation and incubated for 2 h at 37 °C in 5 % CO_2_ in air before washing with PBS and replacement with fresh basal medium. Cells were subsequently cultured and analysed by flow cytometry at defined time points.

### Flow cytometry

Cells for flow cytometry were detached with 0.25 % trypsin/0.02 % EDTA and the subsequent suspension was centrifuged to leave a cell pellet. Primary cells were used immediately post-isolation. Cells were then resuspended in magnetic activated cell sorting buffer (MACS) buffer [(consisting of PBS containing 2 mM EDTA (Alfa Aesar, Heysham, UK) and 0.5 % BSA (Sigma-Aldrich)] and FcR blocking solution (Miltenyi Biotec) before incubation with various antibodies (10 μL per 1 × 10^6^ cells unless stated) in a total volume of 100 μL for 20 min at room temperature in the dark. Following labelling, 900 μL of MACS buffer was added to each sample before centrifugation and resuspension in 500 μL of MACS buffer. Samples were analysed using a BD LSRFortessa flow cytometer running BD FACSDiva software and subsequent data analysis was performed using FlowJo (Tree Star, Ashland, OR, USA). Antibodies used were as follows: CD29-Alexa Fluor 488 (5 μL per 1 × 10^6^ cells), CD31-PE, CD34-FITC, CD44-FITC, CD45-PE, CD56-PE, CD73-PE (2 μL per 1 × 10^6^ cells), CD90-APC, CD105-FITC, CD106-PE, CD146-Alexa Fluor 488 (5 μL per 1 × 10^6^ cells), CD166-PE and TNAP-APC (all Biolegend, San Diego, CA, USA) (detailed in Table 1). 7-AAD (Biolegend) was used as a viability dye. All threshold values for flow cytometry were obtained by analysing both autofluorescence of non-labelled hDPSCs and reactivity of isotype matched controls. Events were gated based on forward and side scatter and colour compensation was performed for each panel.

**Table 1:** Cell surface markers used in co-expression analysis of hDPSCs expressing TNAP; each marker is listed with alternative names, a brief description of cell distribution and a brief explanation of function

Marker	Marker of	Reference
CD29 / Integrin β1	Marker of some haematopoietic cells and stromal cells, involved in cell-cell and cell-matrix adhesion, including binding to fibronectin.	(Hynes 1992)
CD31 / Platelet Endothelial Cell Adhesion Molecule-1 (PECAM-1)	Marker of endothelial cells and some haematopoietic cells. Involved in angiogenesis and as a mechanosensor.	(Newman et al. 1990)
CD34	Haematopoietic stem cell marker, involved in attachment to matrix and stromal cells.	(Krause et al. 1996)
CD44 / Homing Cell Adhesion Molecule (H-CAM)	Adhesion molecule found on many cell types, acts as a hyaluronate receptor.	(Aruffo et al. 1990)
CD45 / Leukocyte Common Antigen (LCA) / Protein tyrosine phosphatase receptor type C (PTPRC)	Marker of many haematopoietic cells, involved in T and B cell receptor-mediated activation.	(Trowbridge and Thomas 1994)
CD56 / Neural Cell Adhesion Molecule (N-CAM)	Found on neural cells, some lymphocytes and skeletal cells, has functions in cell-cell adhesion.	(Sinanan et al. 2004)
CD73 / Ecto-5’-nucleotidase	Expressed by multiple cell types, including some haematopoietic cells, stromal cells, epithelial cells and BMSCs. Catalyses AMP to adenosine and has some cell adhesion functionality.	(Airas et al. 1997; Barry et al. 2001)
CD90 / Thy-1	Marker of some haematopoietic stem cells, fibroblasts and BMSCs. Regulates cell adhesion and T cell activation.	(Ghilzon et al. 1999; Rege and Hagood 2006)
CD105 / Endoglin	Marker of endothelial cells, immune cells and MSCs. Part of the TGF-β receptor complex with a key role in angiogenesis.	(Fonsatti et al. 2003)
CD106 / Vascular Cell Adhesion Molecule-1 (VCAM-1)	Marker of endothelial cells and some BMSCs. Regulates adhesion of mononuclear cells in the blood to vasculature.	(Carter and Wicks 2001)
CD146 / Melanoma Cell Adhesion Molecule (MCAM)	Cell adhesion molecule involved in heterophilic cell-cell interactions. Found on many cell types including endothelium, epithelial cells and MSCs.	(Crisan et al. 2008; Shih 1999)
CD166 / Activated Leukocyte Cell Adhesion Molecule (LCAM)	Found on T cells, monocytes, epithelial cells and BMSCs. Mediates T cell development in the thymus and potentially osteogenic differentiation.	(Bowen et al. 1997; Bruder et al. 1998)

### ALP staining

ALP activity was assessed using the leukocyte alkaline phosphatase kit (Sigma-Aldrich) based on napthol AS-MX phosphate and Fast blue RR salt, performed as per the manufacturer’s instructions. Briefly, monolayer cultures from 3 donors were fixed and subsequently incubated with 0.01 % napthol AS-MX with Fast blue RR salt in deionised water for 30 min in the dark.

### Magnetic cell separation

Cultured hDPSCs were detached with 0.25 % trypsin/0.02 % EDTA treatment and the subsequent suspension was centrifuged to leave a cell pellet. The resulting cells were resuspended in 70 μL MACS buffer, 10 μL FcR blocker and 20 μL TNAP-APC for 15 min at room temperature in the dark before the addition of 900 μL MACS buffer. Cells were centrifuged and resuspended in 70 μL MACS buffer, 10 μL FcR blocker and 20 μL anti-APC microbeads (Miltenyi Biotec) before incubation at 4 °C for 15 min. Labelled cells were centrifuged and resuspended in 1 mL MACS buffer before addition to a magnetised LS MACS column, then columns were washed with 1 mL MACS buffer (×3). Eluted unlabelled cells were collected and designated the TNAP^−^ (negative) fraction. Labelled cells, retained on the column, were isolated by demagnetising the column and washing with 2 mL of MACS buffer and were designated the TNAP^+^ (positive) fraction.

### CFU-f assays

TNAP sorted hDPSCs were counted using a Scepter 2.0 cell counter (Millipore, Billerica, MA, USA), diluted and seeded to 6-well plates at a concentration of 500 cells per well. Cells were cultured for 9 days at 37 °C in 5 % CO_2_ in air before fixation with 10 % (v/v) neutral buffered formalin (Sigma-Aldrich) and staining with 1 % (w/v) methyl violet (VWR, UK) for 30 min before washing and colony counting.

### Trilineage differentiation

hDPSCs sorted to be either TNAP+ or TNAP- were seeded to either 6-well plates at a density of 2.5 × 10^4^ cells/cm^2^ for osteogenic and adipogenic differentiation or to centrifuge tubes at a final count of 2 × 10^5^ total cells for chondrogenic differentiation.

#### Osteogenic differentiation

Plated hDPSCs were cultured for 24 h to allow for adherence and spreading before media was aspirated and non-adherent cells were removed. Cells were then cultured in StemPro Osteogenic Differentiation media (Life Technologies) for 2 weeks at 37 °C in 5 % CO_2_ in air with media changes every 3 days. Then, the culture cells were fixed in 70 % ethanol for 1 h, washed 3 times with dH_2_O and stained with 2 % (w/v) Alizarin Red in dH_2_O (Sigma-Aldrich) pH 4.2 for 10 min. Samples were then washed 5 times with dH_2_O before imaging.

#### Adipogenic differentiation

Plated hDPSCs were cultured for 24 h to allow for adherence and spreading before media was aspirated and non-adherent cells were removed. Cells were then cultured in StemPro Adipogenic Differentiation media (Life Technologies) for 2 weeks at 37 °C in 5 % CO_2_ in air with media changes every 3 days. Then, the culture cells were washed twice in PBS and fixed in 10 % formalin for 10 min before further fixation in fresh 10 % formalin for 1 h. Fixed cells were washed twice with dH_2_O and once with 60 % (v/v) isopropanol in dH_2_O for 5 min before air drying at RT. Cells were stained with Oil Red O working solution [Oil Red O stock solution (0.35 g Oil Red O in 100 mL isopropanol) diluted 3:2 with dH_2_O, passed through a 0.2-μm filter] for 10 min before being washed 4 times in dH_2_O and imaged.

#### Chondrogenic differentiation

Sorted hDPSCs in centrifuge tubes were pelleted by centrifugation at 400*g* for 10 minutes and incubated for 1 h in basal media. Following this incubation, the media was aspirated and replaced with StemPro Chondrogenic Differentiation media (Life Technologies), cells were cultured for 2 weeks at 37 °C in 5 % CO_2_ in air with media changes every 3 days. Then, the culture cells were washed twice in PBS and fixed in 10 % formalin for 10 min before further fixation in fresh 10 % formalin for 1 h. Fixed cells were washed twice with dH_2_O and once with 3 % (v/v) acetic acid in dH_2_O for 3 min before staining with 1 % (w/v) Alcian Blue in 3 % (v/v) acetic acid for 30 min. Samples were then washed 3 times in dH_2_O and imaged.

### Immunofluorescence staining

High (5 × 10^4^ cells/cm^2^) and low (5 × 10^3^ cells/cm^2^) density cell cultures were cultured for 4 days in basal media at 37 °C in 5 % CO_2_ in air before fixation in 70 % (v/v) ethanol for 15 min Fixed samples were permeabilised by incubation with 0.1 % Triton X-100 in PBS for 15 min before washing in PBS and blocking with pre-diluted goat serum (GeneTex, Irvine, CA, USA) for 30 min at 37 °C. Samples were then incubated with primary antibodies at dilutions between 1:50 and 1:250 overnight at 4 °C. Primary antibodies used were anti-cytokeratin 19 (FITC conjugated) (Santa Cruz Biotechnology, Dallas, TX, USA), anti-vimentin, anti-nestin and anti-fibronectin (all Abcam, Cambridge, UK). Non-conjugated primary antibodies were labelled with anti-rabbit IgG Alexa Fluor 488 (Abcam) for 30 min in the dark. Samples were then stained with DAPI for 15 min at room temperature to visualise nuclei before washing in PBS. Samples were then imaged using a Zeiss Axio Vert.A1 running Zen 2012 software (Carl Zeiss, Oberkochen, Germany).

### Statistical analysis

All measurements were performed in at least triplicate and all graphs are displayed as the mean ± SEM. The data were subsequently analysed for Gaussian distribution using the Shaprio–Wilk test, normally distributed data between two separate samples were analysed using a two-sample *t* test and non-parametric data were tested using the Mann–Whitney *U* test. Paired parametric data were tested using a paired *t* test. Statistical analysis was performed in IBM SPSS Statistics v.22 (IBM, Armonk, NY, USA) and *P* values of less than 0.05 were considered significant, 0.01 very significant and 0.001 extremely significant.

## Results

### TNAP is expressed by a sub-set of primary dental pulp cells

**Fig. 1:**
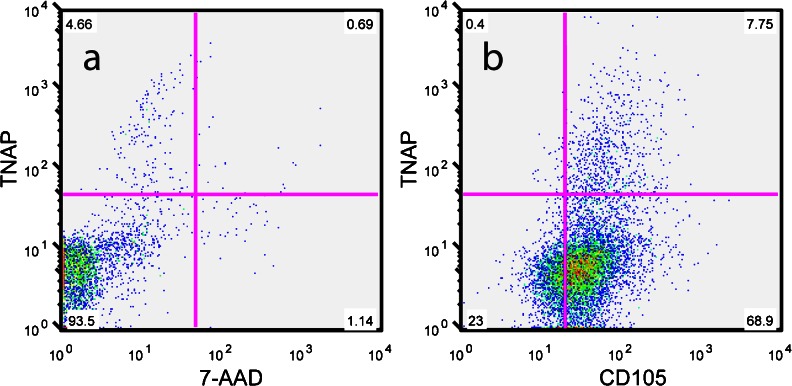
Representative FACS plots showing flow cytometric analysis of TNAP expression by **a** digested human dental pulp with 7-AAD viability dye co-staining and **b** p0 culture expanded adherent hDPSCs with CD105 co-expression. These data confirm TNAP expression in both primary dental pulp and p0 culture expanded adherent hDPSCs

**Fig. 2:**
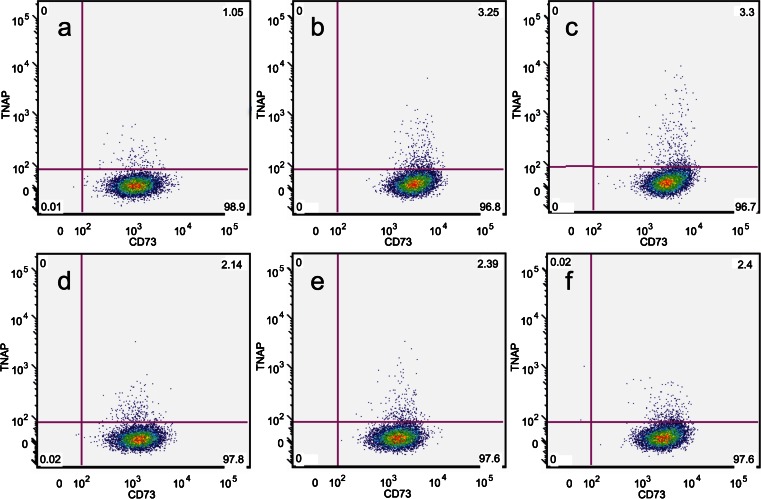
Representative FACS plots showing TNAP/CD73 co-expression across passages from p2 to p7 (**a**–**f**). TNAP was found to be stably expressed throughout culture and over multiple passages it was expressed by what appeared to be a consistent percentage of hDPSCs

Analysis of whole digested dental pulp by flow cytometry confirmed the presence of TNAP+ cells within the primary dental pulp (Fig. 1a). TNAP+ cells were present at ratios between 2 and 10 % of total cells and these values show consistency with the expected number of odontoblasts in primary dental pulp (Murray et al. 2002; Vavpotič et al. 2009). Cell sorting of dental pulp with TNAP did not give proliferative, colony-forming cells (data not shown). Analysis by flow cytometry of adherent p0 hDPSCs, derived from pulp digestion and subsequent colony formation without sorting, showed a subset of cells expressing TNAP, co-expressed with the BMSC marker CD105 (Fig. 1b). This expression was observed on cells derived from 5 donors and was consistently found on between 2 and 8 % of total adherent hDPSCs. Subsequent flow cytometric analysis of cultured hDPSCs over multiple passages, up to p7, showed that TNAP expression was sustained throughout expansion culture (Fig. 2). Expression was found to vary between 1 and 5 % of total cells, with no apparent differences found between expression levels over passage.

### TNAP is co-expressed with multiple markers of hDPSCs

**Fig. 3:**
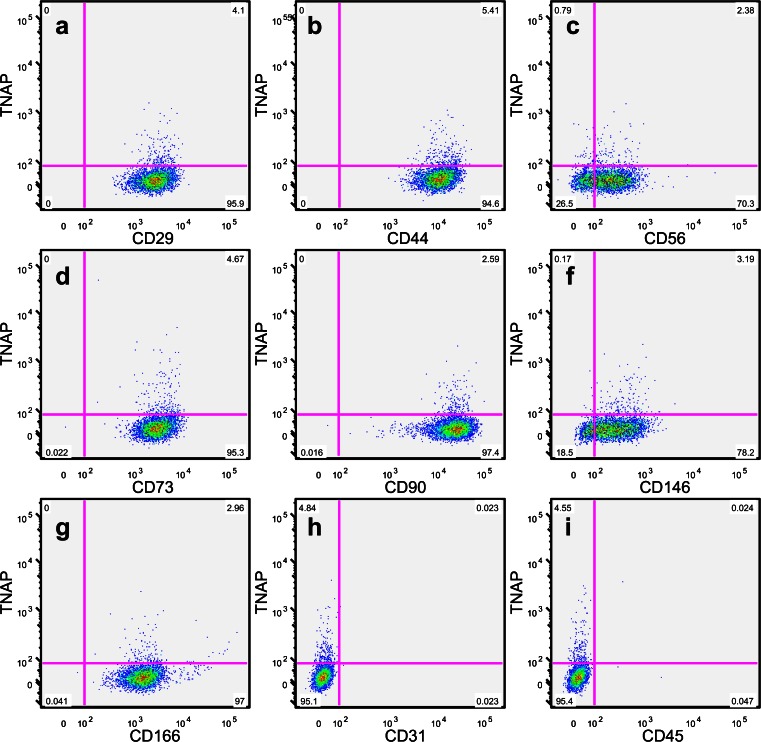
Representative FACS plots showing TNAP co-expression with the common dental stromal cell markers CD29 (**a**), CD44 (**b**), CD56 (**c**), CD73 (**d**), CD90 (**e**), CD146 (**f**) and CD166 (**g**) and the negative control markers CD31 (**h**) and CD45 (**i**). These data show that TNAP is co-expressed with other common markers of hDPSCs and does not map to a separate subpopulation of hDPSCs

Following confirmation of the persistence of TNAP expression, multiple markers of hDPSCs (see Table 1) were analysed to determine their co-expression with TNAP. hDPSC populations were found to be uniformly positive for CD29, CD44, CD73, CD90 and CD166, express CD56 and CD146 to varying degrees and be negative for CD31 and CD45 (Fig. 3). Expression of each marker was found to be independent of TNAP expression, with no difference seen in the expression profile of each marker for TNAP+ or TNAP- hDPSCs.

### Expression of TNAP by hDPSCs is increased by extended periods in culture

**Fig. 4:**
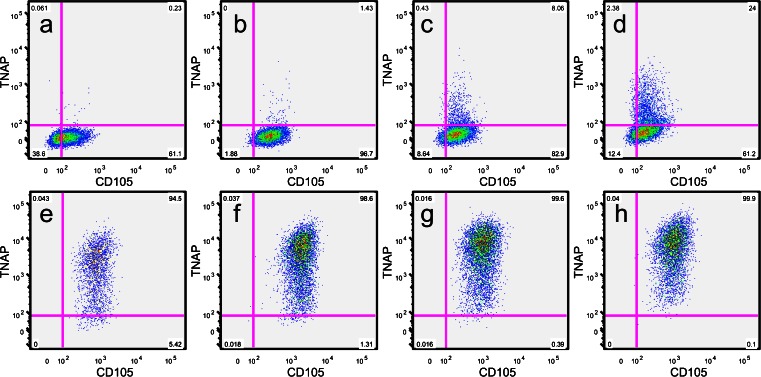
Representative FACS plots showing TNAP expression by hDPSCs (**a**–**d**) and BMSCs (**e**–**h**) over a 14-day time course (**a**, **e** Day 4, **b**, **f** Day 7, **c**, **g** Day 11, **d**, **h** Day 14). All cells were seeded at an initial density of 5 × 10^3^ cells/cm^2^. These data show increasing expression of TNAP over time by both hDPSCs and BMSCs

All the above analyses of hDPSCs investigating initial TNAP expression levels and co-expression were performed on sub-confluent (80–90 % confluency) cell monolayers or initial colonies. However, when hDPSCs were seeded at defined concentrations and cultured for up to 2 weeks without passage, it was found that TNAP expression levels increased with time (Fig. 4). Expression levels were found to be less than 1 % at day 4, steadily rising over time to 26 % of cells by day 14. These findings were in contrast to BMSC controls that had almost uniform expression of TNAP at all timepoints.

### Expression of TNAP by hDPSCs is increased at high cell density

**Fig. 5:**
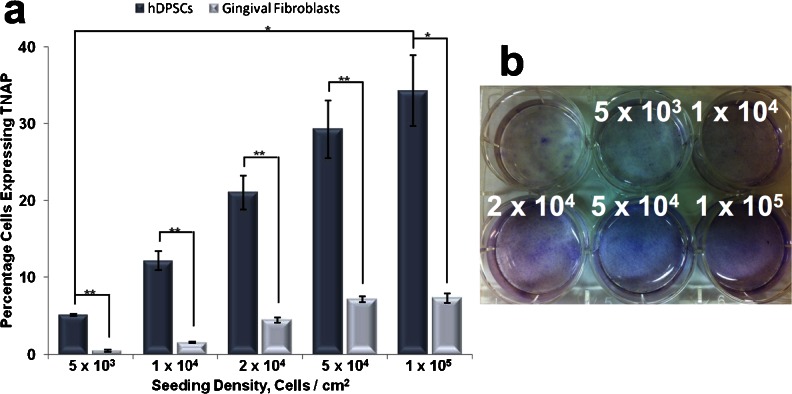
Production of TNAP with increasing cell density. **a** Flow cytometric analysis of TNAP expression by hDPSCs and hGFs with increasing cell density showing the percentage of cells to express TNAP. **b** Cytological staining of TNAP in cell cultures seeded with increasing density using napthol AS-MX and Fast blue RR salt. These data show that TNAP expression increases with increasing cell density. *n* = 5. *p ≤ 0.05, **p ≤ 0.01

Following these findings, we wished to determine whether the observed increase in TNAP+ hDPSCs over time was related to cell density as opposed to time in culture. The effect on TNAP expression of varying hDPSC seeding density was therefore assessed. In these experiments, hGFs were used as a control with low TNAP expression (Somerman et al. 1988). hDPSCs and hGFs were seeded between 5 × 10^3^ cells/cm^2^ and 1 × 10^5^ cells/cm^2^ and cultured for 7 days. TNAP expression was then determined by flow cytometry. At low seeding densities (5 × 10^3^ cells/cm^2^), 5.08 % of hDPSCs were found to express TNAP after 7 days (Fig. 5a) compared to 34.26 % at high seeding densities (1 × 10^5^ cells/cm^2^) (*p* = 0.03). Seeding densities between these values showed a gradual increase in TNAP expression. In comparison, hGFs were found to express TNAP at levels of 0.43 and 7.3 % at comparative seeding densities with a gradual increase of expression observed between these values. Therefore, it can be seen that there is a significant difference between the expression of TNAP on hDPSCs and hGFs at both low (*p* = 0.021) and high (*p* = 0.03) seeding densities. The activity of hDPSC TNAP was confirmed by cytological staining, which showed increased ALP-positive staining with increasing cell density (Fig. 5b).

### Assessment of cytoskeletal proteins

**Fig. 6:**
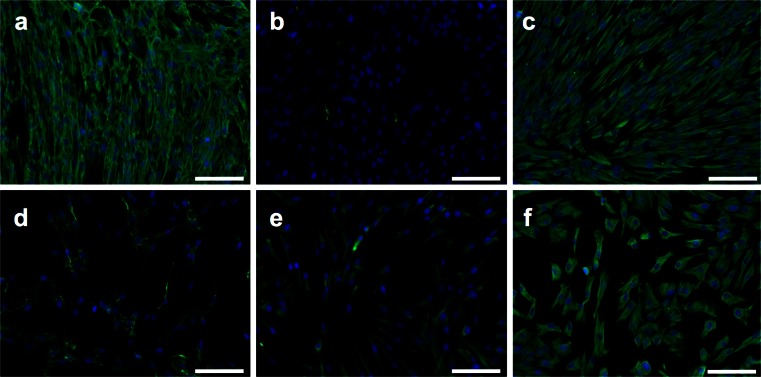
Effect of cell density on cytoskeletal proteins. Immunofluorescence staining (Alexa Fluor 488 secondary antibody) with DAPI nuclear staining showing **a** fibronectin, **b** nestin and **c** vimentin staining of high density (seeded at 5 × 10^4^ cells/cm^2^) hDPSCs. Low density (seeded at 5 × 10^3^ cells/cm^2^) hDPSCs were also stained with **d** fibronectin, **e** nestin and **f** vimentin. Fibronectin appears to be upregulated as hDPSCs increase in density whereas nestin production is reduced. Vimentin production is unaffected by cell density. *Scale bar * 100 μm

Immunofluorescence staining for the cytoskeletal elements vimentin, nestin, fibronectin and cytokeratin 19 in hDPSCs cultured for 4 days at low density (5 × 10^3^ cells/cm^2^) and high density (5 × 10^4^ cells/cm^2^) showed that these cells are positive for vimentin, nestin and fibronectin. Vimentin staining was uniformly positive at both high and low densities while fibronectin production increased with increasing cell density (Fig. 6a, c, d, f). Nestin was present at low cell densities; however, when density increased nestin production was reduced (Fig. 6b, e), cells were negative for cytokeratin 19 at both high and low densities (data not shown).

### Sorting of hDPSCs for TNAP expression

**Fig. 7:**
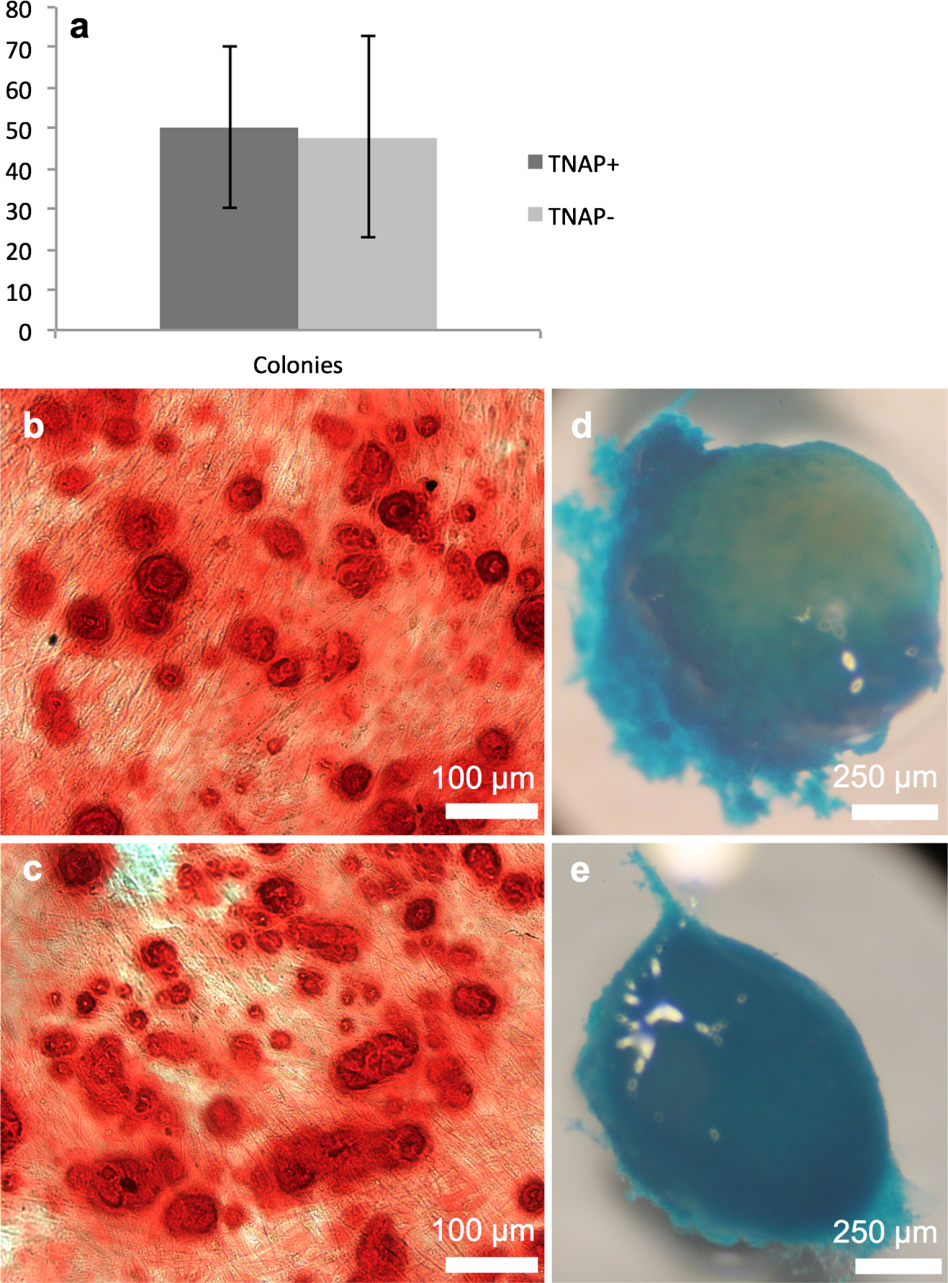
Sorting of hDPSCs for TNAP. **a** Graph showing CFU-f formation by TNAP+ and TNAP- hDPSCs, no significant difference was observed, *n* = 20. **b** Alizarin red staining of osteogenically differentiated TNAP+ hDPSCs; **c** TNAP− cells showing capacity of hDPSCs to mineralize. **d** Alcian blue staining of a chondrogenically differentiated TNAP+ hDPSC pellet; **e** a TNAP– pellet showing GAG production by pelleted hDPSCs. *Scale bar * 100 μm for alizarin red-stained samples, 250 μm for alcian blue-stained pellets

To test whether TNAP+ hDPSCs represent a separate cell population, we employed magnetic cell separation to isolate both TNAP+ (positive) and TNAP− (negative) populations from cultured hDPSCs. These cells were then assessed for colony-forming capacity and differentiation potential. CFU-f assays showed that there was no significant difference in the number of colonies formed by either cell type (*p* = 0.58) although there were on average more colonies present in the TNAP+ fraction (Fig. 7a). Trilineage differentiation experiments showed that both cell populations were capable of in vitro mineralisation, producing strongly Alizarin Red stained mineral deposits across the culture dish, although the deposits in the TNAP+ cells were slightly more uniform across the culture vessel (Fig. 7b, c). The cells from both populations were also capable of producing glycosaminoglycans (GAGs) in in vitro pellet cultures, as shown by Alcian Blue staining (Fig. 7d, e). Neither cell type was able to produce lipid droplets in vitro as determined by Oil Red O staining (data not shown).

### Expression of TNAP by hDPSCs is increased by inhibition of hDPSC proliferation

**Fig. 8:**
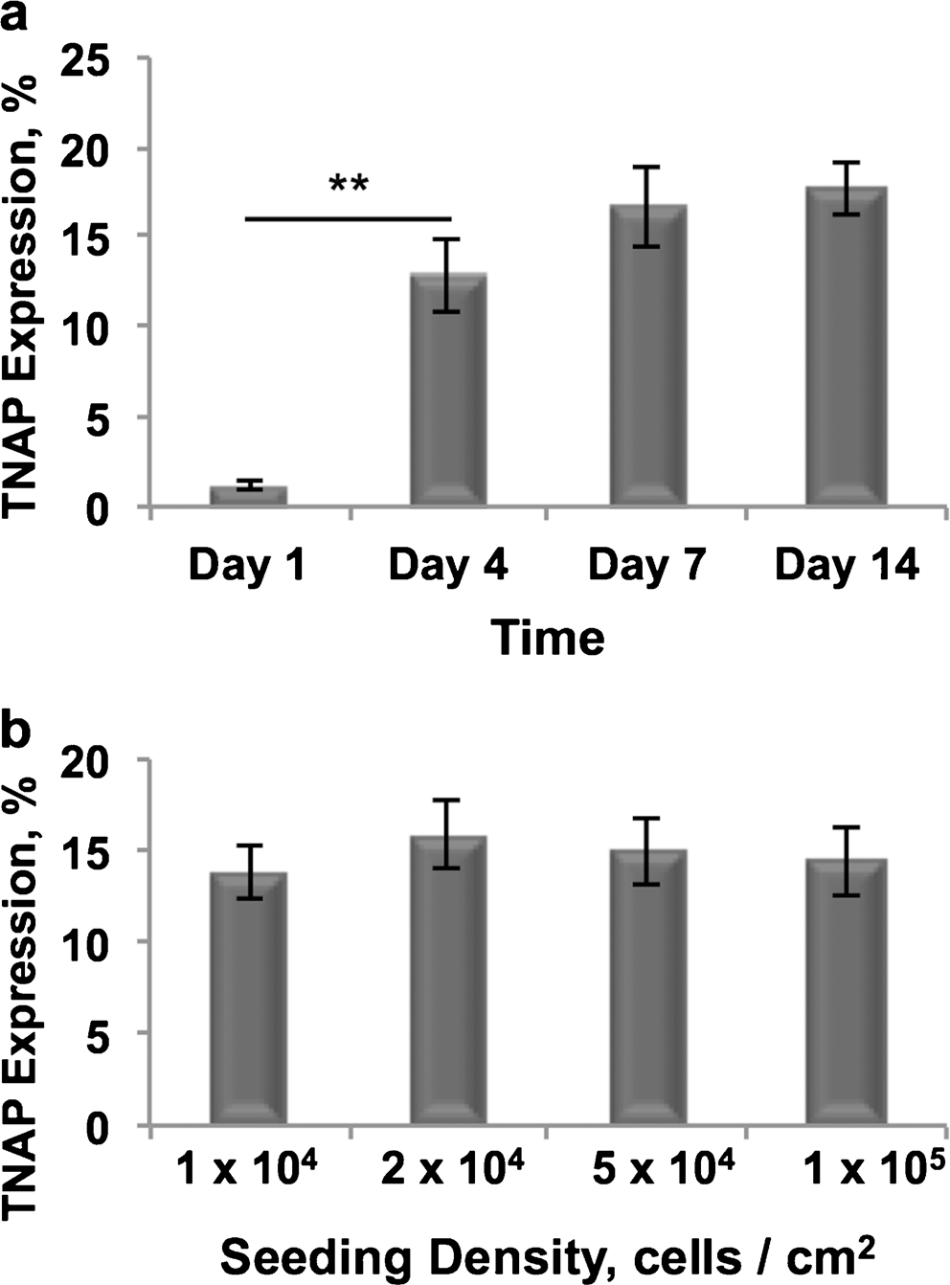
Flow cytometric analysis of TNAP expression by mitotically inactivated hDPSCs seeded at 2 × 10^4^ cells/cm^2^ and cultured for 14 days (**a**) and hDPSCs seeded at varying concentrations and cultured for 7 days (**b**). TNAP expression increased significantly between Days 1 and 4 cultures (*p* = 0.003) and then steadily between Days 4 and 14. No significant differences in TNAP expression were observed when seeding density was varied (*p* = between 0.421 and 0.835). *n* = 6, **p ≤ 0.01

Given that cell proliferation would be expected to be inhibited at the higher cell density values tested, we decided to mimic this effect with mitomycin C treatment in comparatively low density cultures. Following treatment, we measured TNAP expression by mitotically arrested hDPSCs to determine the effect of inhibition of proliferation at several time points and seeding densities. Flow cytometric analysis of TNAP expression by mitomycin C-treated cells revealed that 1.2 % of hDPSCs, seeded at 2 × 10^4^ cells/cm^2^, expressed TNAP 1 day post-treatment and this proportion then rose significantly to 12.8 % after 4 days, (*p* = 0.003) (Fig. 8a). A further rise in TNAP expression from 12.8 to 17.7 % was observed between Days 4 and 14. Assessment of varying seeding densities following mitomycin C treatment showed no difference in TNAP activity after 1 week in culture (*p* values between 0.421 and 0.835) (Fig. 8b).

## Discussion

This study demonstrated that TNAP is present in human dental pulp in vivo and on cultured hDPSCs in vitro; these findings are complementary to previous studies that have shown the expression of TNAP by odontoblasts in vivo and BMSCs in vitro (Battula et al. 2009; Hotton et al. 1999). We also showed that TNAP production in vitro is affected by the density and proliferative ability of the cells in culture and that cytoskeletal markers are also affected by density.

Uncultured, digested human dental pulp found TNAP to be expressed by approximately 2–10 % of cells, which shows consistency with previous findings on odontoblast cell density (Murray et al. 2002; Vavpotič et al. 2009). Cell sorting experiments for TNAP using primary dental pulp did not yield proliferative, adherent cells (data not shown); it is therefore probable that the TNAP positive cells identified in the digested human pulp were not from a stromal cell type. Analysis of colony-derived adherent hDPSCs confirmed the presence of TNAP on a small population of proliferative, adherent cells. However, these were unlikely to contain cells derived from primary odontoblasts due to the difficulty in culturing these cells in vitro (Holt et al. 2001). Taken together, the findings of TNAP presentation by both primary dental pulp and dental pulp-derived adherent cells shows that, whilst TNAP is present on specialised cells in vivo, it appears that it subsequently becomes expressed by more proliferative (and presumably less specialised) cells in vitro.

To confirm that TNAP was expressed by proliferative hDPSCs flow cytometric studies were performed. These studies showed that TNAP was expressed consistently throughout culture from passage 2 to 7 with no apparent differences between expression levels observed. That the expression level of TNAP is static over multiple passages confirms that TNAP is present on proliferating cells, as this proportion would decrease if TNAP+ cells were static.

Following confirmation of the persistence of TNAP expression in cultured hDPSCs, the question of whether TNAP+ cells constituted a separate subpopulation of hDPSCs was examined by investigating TNAP co-expression with multiple known hDPSC markers. The results from these experiments showed that TNAP expression was grouped with the expression of all common hDPSC markers tested; no divergent populations were observed. TNAP is therefore apparently not associated with a unique subpopulation of hDPSCs, or a contaminating cell type and hDPSCs retain their phenotype whilst expressing TNAP. This finding is interesting because it shows that TNAP expression is resident within the hDPSC population, suggesting that a small number of these cells are undergoing phenotypic changes. Further studies with hDPSCs in culture for extended periods showed that over a 14-day period there was a steady increase in TNAP expression with over 26 % of hDPSCs ultimately expressing TNAP. This increase occurred in basal medium in the absence of osteoinductive cues. This is potentially an important point, as TNAP expression is often taken as being evidence of induced osteogenic differentiation (Cheng et al. 1994; Ikeda et al. 2006; Jaiswal et al. 2000). Experiments using osteogenic cultures often measure ALP expression over a period of 21–35 days and potentially there could be an intrinsic increase in TNAP expression over time in culture (Wei et al. 2007; Woods et al. 2009). If hDPSC expression of TNAP is intrinsic to some cells within the population, then this potentially has implications for studies utilising ALP as a marker or other outcome measure following intervention, as in this instance TNAP was present in the absence of osteoinductive cues. It is also possible that osteogenic differentiation is a default pathway for hDPSCs in in vitro culture and that these cells are already in the early stages of differentiation. What both these points highlight is the need to better understand the mechanism of TNAP upregulation in hDPSCs.

TNAP expression seemed to increase more rapidly when the hDPSCs reached confluence, suggesting that TNAP expression might be linked to cell density. We therefore tested the effect of increasing cell density on TNAP expression over a fixed 7-day culture period. Increasing the initial seeding density of hDPSCs in culture caused a significant upregulation in TNAP expression over the time course with a seven-fold increase in TNAP+ expression seen at the highest seeding density. To test whether this was a general pattern of behaviour amongst cells derived from the oral cavity, or a more specific property of hDPSCs, TNAP expression was determined for concurrent cultures of hDPSCs and hGFs at varying seeding densities. These experiments showed that TNAP expression by hGFs follows a similar pattern to hDPSCs with increasing expression observed with increasing cell density. However, the level of TNAP expression by hGFs was significantly lower at all seeding densities, indicating that hDPSCs are more predisposed to increasing TNAP production and therefore potentially more likely to differentiate towards a mineralised phenotype. This apparent predisposition makes TNAP a potentially interesting target for cell sorting in vitro-cultivated hDPSCs for bone tissue engineering applications. In addition, the density-dependent nature of TNAP expression means that this system can potentially be controlled so as to increase expression rapidly. Cell sorting for TNAP expression yielded cells in both fractions that were capable of in vitro mineralisation and GAG production. Neither positive nor negative cells were able to produce lipid droplets under adipogenic conditions and CFU-f assays showed that colonies were formed by both positive and negative cells. What will be of interest for future work will be to determine the sorting criteria necessary to effectively isolate the pro-mineralising cells from dental pulp, as under the regime used in this study there was a higher than expected degree of cell death post-sorting, possibly related to shear sensitivity of the sorted cells and time out of culture. Also, the in vitro mineralisation experiments required a degree of cell expansion to generate a monolayer of cells, which potentially allowed the TNAP− cells time to upregulate TNAP production as the cells became more dense, meaning they had become phenotypically similar to the TNAP+ cells; however, further work will be needed to elucidate this.

Interestingly, cytoskeletal elements were also seen to change in high and low density cultures, with fibronectin production increasing with density, whereas nestin production reduced to barely detectable levels. These data show that large phenotypic changes are affecting the hDPSCs as they become dense in culture. Lineage commitment has been previously shown to be regulated by cell shape and cytoskeletal proteins and our data show that cytoskeletal protein composition in hDPSCs changes as the cells begin to become more dense in culture (Mcbeath et al. 2004). These data indicate that cytoskeletal markers could potentially be used to determine hDPSC lineage status as well as cell surface markers such as TNAP.

hDPSCs upregulate TNAP production when they are at high cell density in vitro. To separate out the effects of cell proliferation on our data, hDPSCs seeded at a medium cell density were treated with mitomycin C to arrest proliferation. The results showed that TNAP expression was significantly increased when mitosis in hDPSCs was blocked and the cells were unable to proliferate. This is potentially analogous to the scenario whereby high density hDPSCs are unable to proliferate due to contact inhibition, although the level of expression is lower, indicating that cell–cell contacts also play an important role in TNAP upregulation. These results indicate that hDPSCs begin to express TNAP when they cannot undergo mitosis and switch from a proliferative state to a differentiating one, making this switch independent of external osteoinductive cues. Indeed, terminal differentiation is often preceded by exit from the cell cycle and a switch from a proliferative phenotype to a differentiating one (Buttitta and Edgar 2007). Studies involving embryonic stem cells have also shown that, in high density cultures, cells will switch from a proliferative state to a differentiating one due to cell–cell contact formation (Reubinoff et al. 2000). Further studies with embryonic stem cells have shown that early markers of osteogenesis, such as osterix, are upregulated at high cell densities, indicating that contact inhibition acts as a cue for the initiation of osteogenic differentiation (Kärner et al. 2009). This is reinforced by high density pellet and monolayer cultures of primary osteoblasts, which have been shown to have enhanced osteogenic gene expression compared to low density monolayer cultures (Jahn et al. 2010).

Overall, this work has confirmed the expression of TNAP (MSCA-1) for hDPSCs but has gone on to show TNAP expression is persistent throughout long-term culture and maps to the proliferating compartment of isolated dental pulp. We have also shown, for the first time, that TNAP expression can be modulated by culturing the cells at varying densities and that increased TNAP expression is likely due to inhibition of proliferation, suggesting that these cells are beginning to undergo lineage commitment in the absence of external environmental cues. TNAP can therefore be seen as a potentially useful marker for regenerative therapies involving hDPSCs.
